# Disparities in years of potential life lost among racial and ethnic groups in Washington state

**DOI:** 10.1186/s13690-022-00969-1

**Published:** 2022-09-21

**Authors:** Solmaz Amiri, Sandte L. Stanley, Justin T. Denney, Dedra Buchwald

**Affiliations:** 1 Institute for Research and Education to Advance Community Health, Elson S. Floyd College of Medicine, Washington State University, Spokane, WA USA; 2 Department of Sociology, Washington State University, Pullman, WA USA

**Keywords:** Premature mortality, Years of potential life lost, Racial and ethnic disparities

## Abstract

**Background:**

The disproportionate mortality burden racial and ethnic groups endure compared to their non-Hispanic white (NHW) counterparts is a widely known public health issue in the United States.

**Methods:**

We examined disparities in premature mortality through a measure of years of potential life lost (YPLL) among racial and ethnic groups after accounting for individual and place-based risk factors. Data were nearly 400,000 geocoded death records from Washington state mortality records from 2011 to 2018. Decedent records included information on marital status and educational attainment at time of death. We linked these records to census tract indicators of rurality and area deprivation based on residential longitude and latitude coordinates at time of death. We conducted censored Poisson regression to test adjusted associations between racial and ethnic identity and YPLL.

**Results:**

Relative to non-Hispanic whites, non-Hispanic blacks, American Indian and Alaska Natives, Asian or other Pacific Islanders, multiracial, and Hispanic decedents had significantly higher rates of YPLL. Controlling for sociodemographic factors reduced but did not eliminate the disparities in YPLL between non-Hispanic whites and other racial and ethnic groups. Controlling for place-based risk factors did not further attenuate differences.

**Conclusions:**

Racial and ethnic minorities suffer disproportionately from premature mortality. Researchers and policy makers must recognize the disproportionate risks to premature mortality and work together to alleviate them through the delivery of better and more accessible targeted services.

## Background

The disproportionate mortality burden racial and ethnic groups endure compared to their non-Hispanic white (NHW) counterparts is a widely known public health issue in the United States [8, 16, 30, 32]. American Indian and Alaska Natives (AIANs) and Blacks experience substantially higher rates of death and premature mortality compared to all other racial and ethnic groups across a wide array of causes [19, 21, 31, 34]. On average, NHWs outlive most racial and ethnic minority groups by nearly 13 years [5]. Second, differences in premature mortality burden are continuously observed between men and women. Men are more likely to die prematurely compared to women and the average national life expectancy among women is 80.5 years while men’s life expectancy is 75.1 years [2]. While entire groups are at risk of dying prematurely, uncovering information on how many years of life they have lost in association with major sociodemographic factors that contribute to this disparity are crucial and cannot be understood from broad estimates of mortality risk.

Social determinants of health such as education, income, and place of residence have been examined as causes that drive mortality risk. Socioeconomic status specifically has been coined a *fundamental* cause of mortality disparities [26]. The theory of fundamental causes states that those who have higher socioeconomic status (SES) are better able to leverage their education and income to access life-saving resources and networks. Lower SES individuals do not have similar access to invaluable resources and higher SES networks and are therefore at higher risk of mortality [4, 22]. Since place of residence is in large part determined by SES, it plays an important role in determining mortality and contributing to racial and ethnic differences in mortality [7]. In terms of premature mortality, we see an unequal distribution of younger deaths across a myriad of causes among those who experience higher socioeconomic deprivation [20]. There are techniques which take age at death and socioeconomic risk factors into account which provides more context to the impact premature mortality has on society.

Premature death has serious economic implications at both state and national levels. This is increasingly important as the youngest of adults in society make up a large portion of the United States work force and their premature mortality negatively impacts the economy. A recent study [32] shows that younger racial and ethnic minorities, such as AIANs age 25–30 years old, were more likely to die prematurely compared to NHWs. Other studies have more explicitly documented the economic benefits of reducing or even eliminating racial and ethnic mortality disparities. For example, a study in Minnesota estimated that eliminating racial disparities in preventable deaths alone would produce 1 to 3 billion dollars in economic savings [24]. These savings occur in increased productivity in the labor market but the benefits to reduced disparities in mortality extend to improved and diversified innovation, entrepreneurship, and political leadership as well.

In this paper, we include two well documented contextual determinants of mortality, rurality and socioeconomic disadvantage. Rurality has negative impacts on individual mortality prospects. Compared to residents of urban areas, rural residents have higher risks of all-cause and cause-specific mortality [12, 14, 15, 27]. Prior to the 1980s, urban dweller areas faced public health crises such as transmission of infectious diseases and higher rates of mortality due to poor infrastructure and population density issues [39]. Currently, however, that trend has changed and evidence suggests that individuals living in rural areas experience what is described as the *rural mortality penalty* or excess risk of death for those who reside in rural areas [6, 29]. The overwhelming majority of Washington state, 30 of the 39 counties, is designated as rural by the state Department of Health [36].

In addition to rurality, area socioeconomic conditions also impact mortality risk for individuals. In particular, the area deprivation index (ADI) [33], captures important elements of socioeconomic deprivation, economic inequality, and disparities in resource availability across geographic space that are robustly linked to mortality prospects. Previous research has shown that racial and ethnic minorities including AIANs, Black, Asian or Native Hawaiian or other Pacific Islander, and multiracial people have higher odds of premature mortality (dying before the age of 65) compared to Whites, and those racial and ethnic minorities residing in deprived areas have double to quadruple the risk of premature cancer mortality than Whites in affluent areas [23].

In the current study, we provide adjusted estimates of the association of racial and ethnic identity with Years of Potential Life Lost (YPLL) for 4 racial and ethnic minority groups, including decedents of multi-racial identity. YPLL, compared to other mortality estimates, is a particularly valuable measure of premature mortality. YPLL compares observed age at death with life expectancy to estimate the average time an individual would have lived had that individual not died relative to the general population’s expected age of death. Compared to other estimates of mortality such as rate ratios or relative risk, YPLL captures loss resulting from mortality in several domains, social and economic, making this measure stand apart in its ability to expose the impact that premature death has on society.

## Methods

### Data

Data on registered deaths for Washington State were obtained from the state Department of Health, Center for Health Statistics for the years 2011–2018 [37]. The data file included information on decedents’ age, sex, race, ethnicity, education, marital status, and residential longitude and latitude. Inclusion criteria for this study were individuals who were 25 years and older at the time of death. People at this age are mature and have a more stable level of education than those 18 and younger [13]. Our final data set includes nearly 400,000 decedents from Washington state over the study period.

### Measures

#### Outcome variable

YPLL is calculated by subtracting the age at the time of death from a predetermined end point age [10]. The premature mortality benchmark of 75 has been used based on U.S., Canadian, Australian, and European studies quantifying the burden of premature mortality [1, 3, 28, 40]. For example, using the end point age of 75, an individual who died at the age of 25 would have 50 years of life lost and those who died at age 75 or above would have 0 YPLL. This approach censored YPLL at zero for decedents whose age at death was 75 or above.

#### Individual-level explanatory variables

Individual-level variables included race and ethnicity (six categories: NHW, NH Black, NH AIAN, NH Asian or Native Hawaiian or other Pacific Islander, NH multiracial (2 or more racial identities), and Hispanics), sex, educational attainment at time of death, and marital status at time of death.

#### Community-level explanatory variables

Rurality was classified using the Rural–Urban Commuting Area (RUCA) codes based on decedents’ residential location at the time of death. RUCA codes use work commuting information, population data, and measures of urbanization to classify urban and rural areas at the census tract level. RUCA codes of 1–3 were classified as metropolitan areas, codes of 4–6 were classified as micropolitan areas, and codes 7 through 10 were classified as small towns and rural areas [36]. The ADI, a validated composite score of socio-economic disadvantage, was used to quantify the social and economic characteristics of census tracts [17, 33]. The ADI was developed based on 17 Census variables in four domains of poverty, housing, employment, and education. We divided ADI scores into terciles of deprivation (1 = least-deprived, 2 = middle-deprived, 3 = most-deprived).

### Statistical analysis

Descriptive statistics included measures of central tendency and variability for continuous variables and frequency distributions and percentages for categorical variables. The Interquartile Range (IQR) will be calculated to determine the midpoint of average death for each racial and ethnic group and determine the spread of death across all groups. We used censored Poisson regression to test the association between race/ethnicity and YPLL controlling for other individual and community-level explanatory variables. This modeling technique was appropriate because we censored YPLL at zero for decedents whose age at death was 75 or above. Preliminary analysis of data showed that a clustering effect was small (intraclass correlation coefficient: 0.01 – 0.03), thus, multilevel estimation was unnecessary.

We start with an adjusted model to examine the relationship between YPLL and race/ethnicity, controlling for sex and year. Next, we progressively adjust for education and marital status of the decedent, and then for residential census tract rurality and area disadvantage. In addition to models on all decedents, we also stratify results and present male and female specific estimates as a priori because average national life expectancy among women and men differs. Associations are presented as incidence risk ratios (IRR) with 95% confidence intervals (CI). Confidence intervals that did not include zero were considered statistically significant. The R-software was used for analysis.

## Results

Table 1 shows characteristics of all decedents age 25 and older in Washington State and also stratified by sex. Deaths with residential locations with address matching accuracy of 80% or above were included in the analyses. Less than two percent of deaths were excluded because they occurred before individuals turned 25. For brevity, we focus on a limited number of characteristics for all decedents here. Decedents were predominantly NHW (88.6%). Deaths were evenly distributed among men and women during the study period.

**Table 1:** Characteristics of decedents age 25 and older in Washington State between 2011 and 2018

Variables	**All Decedents**				**Male Decedents**				**Female Decedents**			
	n	%	Age (mean (SD))	Age (median (IQR))	n	%	Age (mean (SD))	Age (median (IQR))	n	%	Age (mean (SD))	Age (median (IQR))
Race/Ethnicity												
NH White	354,411	88.64	76.48 (14.99)	79 (21)	177,318	88.04	73.82 (15.07)	76 (22)	177,093	89.26	79.14 (14.43)	83 (20)
NH Black	10,266	2.57	67.34 (16.6)	67 (24)	5854	2.91	64.91 (16.02)	65 (21)	4412	2.22	70.57 (16.81)	71 (25)
NH AIAN	5043	1.26	64.26 (16.45)	65 (24)	2676	1.33	62.28 (15.92)	63 (22)	2367	1.19	66.5 (16.76)	68 (24.5)
NH Asian or NHOPI	16,250	4.06	73.82 (16.18)	77 (22)	7798	3.87	71.1 (16.43)	74 (23)	8452	4.26	76.33 (15.53)	80 (21)
NH Multi-racial	3318	0.83	64.74 (18.19)	66 (27)	1770	0.88	62.18 (17.87)	63 (26)	1548	0.78	67.68 (18.12)	69 (27)
Hispanic	10,522	2.63	65.2 (18.45)	66 (29)	5996	2.98	62.09 (18.21)	63 (28)	4526	2.28	69.33 (17.95)	72 (27)
Sex												
Female	198,398	49.62	78.36 (14.9)	82 (21)								
Male	201,412	50.38	72.85 (15.57)	75 (22)								
Education												
No high school diploma	58,629	14.66	76.53 (16.1)	81 (22)	29,346	14.57	73.45 (16.68)	77 (24)	29,283	14.76	79.63 (14.88)	83 (18)
High school diploma or equivalent	158,224	39.57	75.8 (15.65)	79 (22)	73,370	36.43	71.56 (16.06)	74 (23)	84,854	42.77	79.46 (14.3)	83 (19)
Some college or associate degree	102,381	25.61	73.57 (15.64)	75 (23)	51,040	25.34	70.88 (15.23)	72 (21)	51,341	25.88	76.23 (15.58)	79 (23)
Bachelor’s degree and above	80,576	20.15	77.04 (14.24)	80 (20)	47,656	23.66	76.57 (13.69)	79 (19)	32,920	16.59	77.73 (14.97)	81 (21)
Marital status												
Never married	34,673	8.67	57.19 (18.04)	57 (25)	23,279	11.56	55.41 (17.08)	56 (24)	11,394	5.74	60.84 (19.36)	60 (28)
Married or living with domestic Partner	152,781	38.21	73.25 (13.47)	75 (19)	101,103	50.20	74.37 (13.26)	76 (18)	51,678	26.05	71.05 (13.59)	72 (20)
Divorced or separated	75,113	18.79	69.3 (13.61)	69 (19)	38,697	19.21	67.14 (12.8)	67 (17)	36,416	18.36	71.59 (14.07)	72 (20)
Widowed	137,243	34.33	86.28 (9.29)	88 (11)	38,333	19.03	85.18 (9.6)	87 (11)	98,910	49.85	86.7 (9.13)	88 (11)
RUCA												
Metropolitan	338,114	84.57	75.53 (15.63)	79 (23)	169,291	84.05	72.68 (15.74)	75 (22)	168,823	85.09	78.39 (14.97)	82 (21)
Micropolitan	34,969	8.75	76.6 (14.81)	79 (21)	17,658	8.77	74.34 (14.84)	77 (21)	17,311	8.73	78.89 (14.43)	82 (20)
Small town or rural	26,727	6.68	74.94 (14.6)	77 (20)	14,463	7.18	72.99 (14.34)	75 (20)	12,264	6.18	77.25 (14.57)	80 (21)
Area-deprivation index												
Less deprived	137,210	34.32	76.93 (15.25)	80 (22)	69,156	34.34	74.22 (15.41)	77 (21)	68,054	34.30	79.67 (14.57)	83 (20)
Middle deprived	136,726	34.20	75.75 (15.31)	79 (22)	68,741	34.13	73 (15.45)	75 (22)	67,985	34.27	78.54 (14.64)	82 (20)
Most deprived	125,874	31.48	73.94 (15.81)	76 (24)	63,515	31.53	71.19 (15.73)	73 (23)	62,359	31.43	76.75 (15.39)	80 (22)

*NH* Non-Hispanic, *AIAN* American Indian or Alaska Native, *NHOPI* Native Hawaiian or other Pacific Islander, *RUCA* Rural–urban commuting codes, *IQR* Interquartile range

Among all decedents, the mean age at death for NHWs (76) was over a decade longer than that of AIAN (64), multi-racial (65), and Hispanic (65) decedents. More consistent with the literature and because the age at death was not normally distributed, we also show the median age at death in Table 1. Large differences exist here as well. Median age of NHWs was 79 (IQR: 21), while the median age of NH Blacks, NH AIANs, multiracial individuals, and Hispanics were 67 (IQR range: 23), 65 (IQR: 24), 66 (IQR: 27), and 66 (IQR: 29), respectively.

Most decedents lived in metropolitan census tracts (84.5%); roughly 9% lived in micropolitan areas and 7% lived in small towns or rural areas. Less than four percent (3.9%) of deaths were excluded because of missing or inaccurate residential address data. Although differences in median age at death across metropolitan (79), micropolitan (79), and rural (77) neighborhoods were minimal the median age of decedents was higher in less-deprived neighborhoods compared to their peers in the most-deprived neighborhoods (80 [IQR = 22] vs. 76 [IQR = 23].

Table 2 shows the results of censored Poisson regression models on YPLL for decedents. In unadjusted models, compared to NHWs, NH Blacks and NH AIANs had, respectively, a rate 1.25 (95% CI = 1.24 – 1.26) and 1.34 (1.33 – 1.35) times higher for YPLL. The rate for NH Asian or Other Pacific Islander decedents was 1.15 (1.14 – 1.15) times higher and for Multiracial and Hispanic decedents 1.46 (1.45 – 1.47) and 1.49 (1.48 – 1.50) times higher compared to NHWs (Model 1).

**Table 2:** Censored Poisson Regression model of associations between race/ethnicity and years of potential life lost among all decedents 25 years and older at the time of death in Washington State between 2011 and 2018

	**All Decedents (*n* = 399,810)**						**Male Decedents (*n* = 201,412)**						**Female Decedents (*n* = 198,398)**					
	Model 1		Model 2		Model 3		Model 4		Model 5		Model 6		Model 7		Model 8		Model 9	
	IRR	CI	IRR	CI	IRR	CI	IRR	CI	IRR	CI	IRR	CI	IRR	CI	IRR	CI	IRR	CI
Year	0.99	0.99 – 0.99	0.99	0.98 – 0.99	0.99	0.98 – 0.99	0.99	0.99 – 0.99	0.99	0.98 – 0.99	0.98	0.98 – 0.99	0.99	0.99 – 0.99	0.98	0.98 – 0.99	0.98	0.98 – 0.99
Race/Ethnicity																		
NH White (reference)																		
NH Black	1.25	1.24 – 1.26	1.13	1.13 – 1.14	1.12	1.12 – 1.13	1.28	1.27 – 1.29	1.14	1.13 – 1.16	1.13	1.12 – 1.15	1.23	1.23 – 1.24	1.13	1.12 – 1.14	1.12	1.11 – 1.13
NH AIAN	1.34	1.33 – 1.35	1.21	1.20 – 1.22	1.22	1.21 – 1.23	1.35	1.34 – 1.37	1.27	1.25 – 1.28	1.26	1.25 – 1.28	1.34	1.32 – 1.35	1.18	1.16 – 1.19	1.19	1.18 – 1.21
NH Asian or NHOPI	1.15	1.14 – 1.15	1.18	1.17 – 1.19	1.17	1.16 – 1.18	1.14	1.13 – 1.15	1.16	1.15 – 1.17	1.16	1.15 – 1.17	1.15	1.14 – 1.16	1.20	1.19 – 1.21	1.18	1.17 – 1.19
NH Multi-racial	1.46	1.45 – 1.47	1.35	1.34 – 1.36	1.34	1.33 – 1.36	1.44	1.42 – 1.46	1.36	1.34 – 1.38	1.36	1.34 – 1.38	1.47	1.46 – 1.49	1.34	1.32 – 1.36	1.33	1.32 – 1.35
Hispanic	1.49	1.48 – 1.50	1.39	1.39 – 1.40	1.39	1.39 – 1.40	1.43	1.42 – 1.45	1.36	1.35 – 1.38	1.36	1.34 – 1.37	1.52	1.51 – 1.53	1.41	1.40 – 1.42	1.41	1.4 – 1.42
Sex																		
Female (reference)																		
Male	1.08	1.08 – 1.09	0.99	0.99 – 0.99	0.99	0.99 – 0.99	–		–		–		–		–		–	
Marital status																		
Married / cohabitating (reference)																		
Never married			1.81	1.81 – 1.82	1.80	1.80 – 1.81			1.67	1.66 – 1.68	1.66	1.65 – 1.67			1.88	1.87 – 1.89	1.87	1.87 – 1.88
Divorced or separated			1.07	1.07 – 1.07	1.07	1.06 – 1.07			1.00	0.99 – 1.00	0.99	0.99 – 1.00			1.12	1.11 – 1.12	1.11	1.11 – 1.12
Widowed			0.62	0.61 – 0.62	0.61	0.61 – 0.62			0.58	0.57 – 0.58	0.57	0.57 – 0.58			0.67	0.66 – 0.68	0.67	0.66 – 0.68
Education																		
No high school diploma (reference)																		
High school diploma or equivalent			1.00	1.00 – 1.01	1.00	1.00 – 1.01			0.99	0.98 – 0.99	0.99	0.98 – 1.00			1.01	1.01 – 1.02	1.01	1 – 1.01
Some college or associate degree			1.00	1.00 – 1.01	1.00	1.00 – 1.01			1.03	1.02 – 1.04	1.03	1.03 – 1.04			0.99	0.99 – 1.00	0.99	0.98 – 0.99
Bachelor’s degree and above			0.89	0.89 – 0.9	0.89	0.89 – 0.9			0.95	0.94 – 0.95	0.96	0.95 – 0.96			0.86	0.86 – 0.87	0.86	0.85 – 0.86
RUCA																		
Metropolitan (reference)																		
Micropolitan					0.94	0.94 – 0.95					0.95	0.94 – 0.96					0.94	0.93 – 0.94
Small town or rural					0.91	0.90 – 0.91					0.92	0.91 – 0.93					0.89	0.89 – 0.9
Area-deprivation index																		
Less deprived (reference)																		
Middle deprived					1.01	1.01 – 1.01					1.02	1.01 – 1.02					1.00	1.00 – 1.01
Most deprived					1.03	1.02 – 1.03					1.06	1.05 – 1.07					1.01	1.00 – 1.01

*NH* Non-Hispanic, *AIAN* American Indian or Alaska Native, *NHOPI* Native Hawaiian or other Pacific Islander, *RUCA* Rural–urban commuting codes, *IRR* Incidence risk ratio, *CI* Confidence interval

Estimates for the relationship between YPLL and race/ethnicity (except for NH Asian or Other Pacific Islander) were attenuated across Model 1 and 2. For example, adjusting for sex, marital status and educational attainment in Model 2 results in an IRR for NH Blacks of 1.13 (1.13 – 1.14), down from 1.25 in Model 1; a 48% lower ([1.25–1.13] / [1.25–1.00] * 100) IRR. Importantly, although the IRRs for racial and ethnic minorities are lower in Model 2, compared to Model 1, they remain higher than NHWs. Estimates for the relationship between YPLL and race/ethnicity remained stable across Model 2 and 3. Further adjusting by RUCA and ADI, NH AIANs had a fully adjusted rate 1.22 (1.21 – 1.23) times higher for YPLL compared to NHWs. For NH Blacks and NH Asian or Other Pacific Islander decedents, the rate was 1.12 (1.12 – 1.13) and 1.17 (1.16 – 1.18) times higher, respectively. Multi-racial decedents had a rate 1.34 (1.33 – 1.36) times higher and Hispanics 1.39 (1.39 – 1.40) times higher compared to NHWs (Model 3). Similar patterns were observed in sex stratified models when the results of unadjusted and adjusted models were compared within each sex.

The control variables in these fully adjusted models demonstrate that never married decedents had IRRs 1.81 times higher than married or cohabiting decedents (Model 3); for males this was 1.66 times higher (Model 6) and for females 1.87 times higher (Model 9). Decedents with a bachelor’s degree or more had 0.89 times the rate of YPLL as decedents with no high school diploma. This protection was present for male (IRR = 0.96, Model 6) and female decedents (IRR = 0.86, Model 9) with a bachelor’s degree or more. For all, female and male decedents, living in micropolitan and small town or rural neighborhoods carried lower rates of YPLL, compared to decedents in metropolitan neighborhoods. Lastly, the ADI illustrates that the more disadvantaged the neighborhood the higher the rate of YPLL for all and female decedents but not for male decedents.

## Discussion

The burden of premature mortality is widely understood as being a prominent disparity among racial and ethnic groups. Within Washington State we found that all decedents who identified as racial and ethnic minorities had higher rates of YPLL compared to NHWs—including NH Blacks, NH AIANs, NH Asian or Native Hawaiian or other Pacific Islander, NH multi-racial, and Hispanic decedents. Multiracial and Hispanic decedents had the highest rates of YPLL compared to NHWs even after controlling for important individual and place-based risk factors for mortality. Although the life expectancy of the average person has increased over the last several decades, mortality disparities continue to plague racial and ethnic minorities.

Social, economic, and contextual determinants of health such as marital status, individual educational attainment, rurality, and area-level socioeconomic deprivation all contribute to YPLL and can be addressed through effective social and economic policies. In our study, adjusting for sociodemographic characteristics such as marital status and educational attainment attenuated the relationship between race/ethnicity and YPLL and RUCA and ADI did not have much of an effect on YPLL differences. Disparities in YPLL among racial and ethnic groups remain a significant issue and may be explained by other mechanisms underlying racial and ethnic differences. For example, a related study on premature mortality which explores birth cohort effects among Black and NHW individuals that died between 1960–2009 found differences between Black age cohorts who did and did not endure Jim Crow era policies [18]. This study serves as an example of a time period which helps explain an improvement in premature mortality risk. Could there be something in our data that could lend itself to explaining why sociodemographic factors show no difference between our models? Could there be policies in Washington State that could impact racial and ethnic groups and explain some of the differences in YPLL?

Using YPLL may have illuminated a different context under which race matters for reasons beyond SES. Previous studies have shown that context matters and alternative methods and measures such as exploring particular causes of death instead of mortality generally or the effect of historical context amongst particular groups could help further illuminate why race is so important. Future research must address structural racism that restricts access to affordable housing and economic opportunity, prevents equitable political representation, and hampers attaining good health and well-being [11, 38]. Improvements in risk factors for premature death, such as access to quality education, hold promise to close the gaps in length of life by racial and ethnic identity. Nonetheless, other contributors to racial and ethnic minority gaps in longevity remain to be explored.

These analyses have several notable limitations. First, racial misclassification, typically as white, is a limitation of the data we used in this report. For example, AIAN deaths are undercounted by at least 38% nationally [9, 35], especially on death certificates, the source of our data. Second, we only had data on a single timepoint of decedents’ characteristics such as marital status, residential history, and area deprivation. These characteristics may vary over time and can potentially be risk factors for premature mortality. Third, the majority of decedents in our study were NHW, reflecting the demographics of Washington State residents. Our findings may not necessarily reflect patterns in other states with more diverse populations. Fourth, these data do not include deaths from the novel coronavirus, which has been shown to disproportionately infect and kill by race and ethnicity [25].

## Conclusion

In summary, despite increasing life expectancy and decreases in the gap in life expectancy between whites and racial and ethnic minority groups, important differences in the mortality experience by racial and ethnic identity remain unexplored. Our findings showed that racial and ethnic minorities continue to experience a disproportionate burden of premature death in Washington state. Researchers and policy makers must recognize the disproportionate risks to premature mortality and work together to alleviate them through the delivery of better and more accessible targeted health services.

## Data Availability

Data on all decedents were purchased from the Washington Department of Health. A request to purchase records can be made at https://www.doh.wa.gov/DataandStatisticalReports/WashingtonTrackingNetworkWTN/DataPortal.
